# ATOH8 is crucial for the differentiation of human trophoblast stem cells into extravillous trophoblasts

**DOI:** 10.1038/s41598-025-22484-3

**Published:** 2025-11-04

**Authors:** Joudi Salamah, Mohamed Salamah, Bum-Kyu Lee

**Affiliations:** Department of Biomedical Sciences, Cancer Research Center, College of Integrated Health Sciences, University at Albany, State University of New York, Rensselaer, NY 12144 USA

**Keywords:** ATOH8, EVT differentiation, Trophoblast stem cells, Trophoblast lineage differentiation placental development, Developmental biology, Stem cells

## Abstract

The transcription factor ATOH8 regulates cell fate and differentiation during development. Loss of ATOH8 leads to defects in murine placenta development, yet its specific functions in self-renewal and differentiation of human trophoblast stem cells (TSCs) remain poorly understood. Here, we reveal that ATOH8 is critical for extravillous trophoblasts (EVTs) formation while being dispensable for the self-renewal of TSCs. We show predominant ATOH8 expression in EVTs compared to syncytiotrophoblasts (STs) and TSCs. Knockdown (KD) of ATOH8 in TSCs does not alter their morphology, proliferation, or self-renewal marker expression, indicating that ATOH8 is not required for TSC maintenance. However, during EVT differentiation, ATOH8 expression gradually increases and its depletion results in pronounced morphological abnormalities, impaired expression of EVT markers, sustained TSC marker expression, and abolished invasive capacity. Conversely, ATOH8 overexpression (OE) under self-renewing conditions modestly induces EVT markers, whereas its OE during ST differentiation disrupts ST formation by reducing cell fusion and aberrantly activating EVT genes. Transcriptomic profiling reveals that the loss of ATOH8 during EVT differentiation disrupts pathways critical for placental development, including extracellular matrix organization and PI3K-AKT signaling. We also show that ATOH8 functions within a cooperative network of EVT regulators, reciprocally regulating their expression and maintaining a transcriptional circuit essential for EVT specification. Collectively, these findings establish ATOH8 as an indispensable regulator of EVT differentiation and invasive function, orchestrating EVT-specific gene programs and pathways alongside other key transcription factors to ensure proper EVT formation.

**Supplementary Information:**

The online version contains supplementary material available at 10.1038/s41598-025-22484-3.

## Introduction

The placenta, a temporary organ that forms in the uterus during pregnancy, is crucial for a healthy pregnancy, functioning as a dynamic interface between mother and fetus. It ensures a healthy pregnancy by facilitating oxygen and nutrient transfer, eliminating waste, secreting essential hormones, and providing physical and immunological protection^1^. Disruptions in placental development and functions increase susceptibility to infections and can lead to various complications, including fetal hypoxia, intrauterine growth restriction (IUGR), preeclampsia (PE), stillbirth, and miscarriage, jeopardizing organ development and fetal survival^2^. Beyond pregnancy, placental health has lifelong implications. Placental insufficiency can program the fetus for increased risk of various chronic adult diseases, such as type 2 diabetes, hypertension, cardiovascular diseases, and psychiatric disorders in later life^3–5^. Thus, the placenta is not only central to successful gestation but also a key determinant of long-term health, underscoring its irreplaceable role in both immediate pregnancy outcomes and future well-being.

The human placenta consists of specialized trophoblasts, originating from the trophectoderm of the blastocyst, which differentiate into specialized subtypes critical for placental development and function. These include cytotrophoblasts (CTs), syncytiotrophoblasts (STs), and extravillous trophoblasts (EVTs)^6^. CTs act as progenitor cells, maintaining trophoblast renewal and differentiation into STs and EVTs. ST, a multinucleated layer that directly interfaces with maternal blood, facilitates maternal–fetal exchange of oxygen, nutrients, and waste while secreting pregnancy-sustaining hormones. EVTs invade the maternal decidua and remodel spiral arteries to optimize placental blood flow to the placenta^6^. Dysregulation of EVT differentiation or function is implicated in severe pregnancy complications, such as PE, IUGR, and miscarriage, due to impaired vascular remodeling^7,8^. Additionally, trophoblasts mediate immune tolerance by suppressing maternal immune responses to fetal antigens, preventing rejection^9^. Understanding trophoblast lineage specification and EVT invasion mechanisms is therefore fundamental to unraveling the etiology of placental disorders and developing therapeutic interventions to improve pregnancy outcomes.

EVT differentiation is governed by a tightly regulated transcriptional network that controls invasion, immune tolerance, and vascular remodeling during placentation^10–12^. A few transcription factors (TFs) have been reported as EVT regulators, including DLX3, DLX5, ASCL2, SNAI1, EPAS1, and TCF4^11,13–15^. The basic helix-loop-helix (bHLH) transcription factor ATOH8, which exhibits remarkable evolutionary conservation across species^16^, demonstrates pleiotropic functions in both developmental and physiological contexts^17^. During embryogenesis, ATOH8 serves as a critical determinant of cell fate specification and lineage differentiation across multiple organ systems, including the central nervous system, renal tissue, pancreatic development, and retinal formation^18–21^. Beyond development, ATOH8 participates in iron homeostasis maintenance and exhibits tumor suppressor activity, with loss-of-function mutations promoting stem-like properties in hepatocellular carcinoma^22^. Furthermore, ATOH8 modulates cellular responses to hypoxic stress through direct interaction with hypoxia-inducible factors^17^. In the context of placental development, murine knockout models have established ATOH8 as an essential regulator of early placental development, where it coordinates decidual vascularization and extracellular matrix reorganization^23^. It plays a pivotal role in controlling trophoblast behavior at the maternal–fetal interface, governing their invasion capabilities, proliferative activity, and differentiation potential. Through these coordinated actions, ATOH8 ensures the establishment of a functional placental architecture necessary for successful pregnancy. While ATOH8 has been demonstrated to participate in human endometrial stromal fibroblast decidualization^24^, an essential preparatory step for placental development, its specific functional contributions to human trophoblast biology remain to be fully elucidated.

In this study, we observed an upregulation of ATOH8 expression during TSCs differentiation into EVTs. Loss-of-function and gain-of-function studies demonstrated that ATOH8 is dispensable for TSC self-renewal but essential for EVT differentiation and invasion. In contrast, overexpression (OE) of ATOH8 impaired ST differentiation, whereas its OE in TSCs is not sufficient to induce EVT differentiation. These results elucidate the critical role of ATOH8 in EVT differentiation and contribute novel insights into its function in human placental development.

## Results

### ATOH8 is not required for the self-renewal of human TSCs

**Fig. 1:**
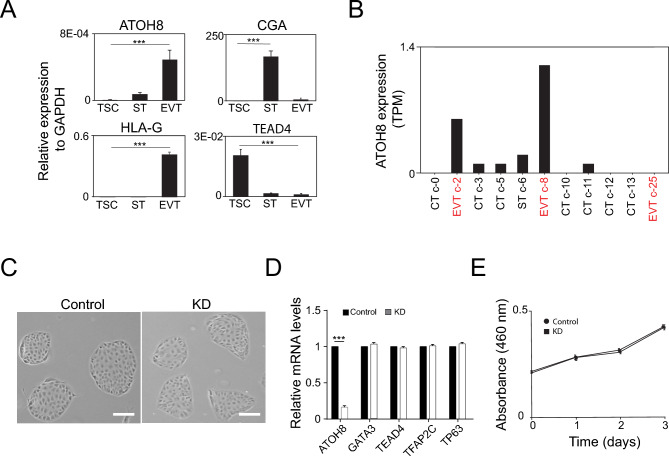
ATOH8 is not required for the self-renewal of human TSCs. (**A**) Relative mRNA expression levels of ATOH8, TEAD4 (a TSC marker), CGA (an ST marker), and HLA-G (an EVT marker) to GAPDH, in TSCs, STs, and EVTs. Error bars represent mean ± SD (standard deviation) from three independent biological replicates. Significance between experimental groups was determined using a two-tailed Student’s *t* test. Significance level of *P* < 0.005 is denoted by ***. (**B**) In vivo expression profiles of ATOH8 across various trophoblast subtypes, as determined by single-cell RNA sequencing. Data were obtained from the Human Protein Atlas website. (**C**) Bright-field microscopy images comparing control TSCs and ATOH8-KD TSCs. Scale bars indicate 100 µm. (**D**) Relative mRNA expression of TSC marker genes in ATOH8-KD TSCs compared to control TSCs. Error bars represent mean ± SD (n = 3). Significance between experimental groups was determined using a two-tailed Student’s t-test. Significance level of *P* < 0.005 is denoted by ***. (**E**) Line graph depicting the proliferation rates of control and ATOH8-KD TSCs over time. Error bars represent mean ± SD (n = 3).

Previously, we performed a comprehensive bulk RNA sequencing analysis on human TSCs, TSC-derived STs, and TSC-derived EVTs^11^. This investigation revealed that ATOH8 was most highly expressed in EVTs among those trophoblasts. To substantiate this observation, we assessed ATOH8 expression across these cell types using RT-qPCR and confirmed its predominant expression in EVTs, with minimal expression detected in STs and TSCs (Fig. 1A). Supporting this, single-cell RNA sequencing data analysis consistently demonstrated that EVTs exhibited the highest levels of ATOH8 expression among various trophoblast subtypes, validating its in vivo expression in EVTs (Fig. 1B). In contrast, ST and CT showed comparatively lower ATOH8 levels.

To elucidate the functional significance of ATOH8 in TSCs, particularly its role in their maintenance and self-renewal, we employed RNA interference using short hairpin RNA (shRNA) to deplete ATOH8 expression. As depicted in Fig. 1C, the depletion of ATOH8 did not result in noticeable morphological alterations in TSCs. Correspondingly, quantitative analysis of TSC marker gene expression showed no significant differences between ATOH8-depleted TSCs and control TSCs (Fig. 1D). Additionally, cell proliferation assays revealed that the proliferation rate of ATOH8-depleted TSCs was comparable to that of control TSCs (Fig. 1E). Collectively, these findings indicate that ATOH8 is not essential for the self-renewal of TSCs.

### ATOH8 is essential for EVT formation

**Fig. 2:**
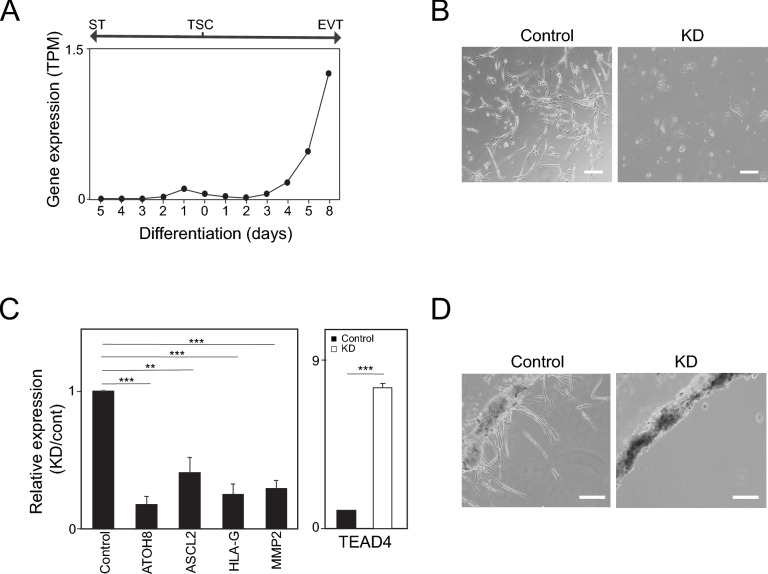
ATOH8 is indispensable for EVT formation. (**A**) Time-course analysis of ATOH8 expression during the differentiation of TSCs into STs and EVTs. (**B**) Bright-field images of control and ATOH8-KD cells following 8 days of differentiation into EVTs (ATOH8-KD EVTs). Scale bars indicate 100 µm. (**C**) Relative mRNA expression of EVT and TSC marker genes in ATOH8-KD EVTs compared to control EVTs. Error bars represent mean ± SD from three independent biological replicates. Significance between experimental groups was determined using a two-tailed Student’s t-test. Significance levels of *P* < 0.01 and *P* < 0.005 are denoted by ** and ***, respectively. (**D**) Bright-field images demonstrating the invasive capacity of control and ATOH8-KD cells after 8 days of EVT differentiation. Scale bars indicate 100 µm.

To characterize the temporal dynamics of ATOH8 expression during TSC differentiation, we conducted a time-course analysis of ATOH8 transcript levels as TSCs differentiated into STs and EVTs. Our analysis showed that ATOH8 expression gradually increased during EVT differentiation, whereas its levels remained relatively unchanged during ST formation (Fig. 2A). This gradual induction suggests that ATOH8 may play a role in activating EVT-specific transcriptional programs. To determine the necessity of ATOH8 in TSC differentiation into EVTs, we used shRNA-mediated knockdown (KD) of ATOH8 in TSCs, followed by the induction of EVT differentiation over an 8-day period. As illustrated in Fig. 2B, ATOH8-depleted cells exhibited profound morphological abnormalities compared to control EVTs, which developed characteristic elongated and protrusive structures associated with invasive behavior. Consistent with these observations, ATOH8 depletion notably reduced the expression of EVT-specific markers while sustaining high levels of the TSC marker TEAD4 (Fig. 2C). This persistent TEAD4 expression in ATOH8-depleted cells indicates that the loss of ATOH8 hinders the proper transition from a stem cell state to a differentiated EVT state. We next assessed whether ATOH8-KD cells retained proliferative capacity after differentiation. Following 8 days of differentiation toward EVT, the cells were harvested and replated in TSC culture medium. Although ATOH8-KD cells showed abnormal morphology (Supplementary Fig. 1A) and grew more slowly (Supplementary Fig. 1B) compared to TSCs, they kept proliferating, proving that they did not terminally differentiate. Therefore, ATOH8 is necessary for TSCs to fully differentiate into EVT.

Given that a hallmark of EVT function is its invasive capability, we assessed the effect of ATOH8 depletion on this property. Invasion assay revealed that ATOH8 depletion severely compromised the invasive capacity of EVTs (Fig. 2D). Together, our findings demonstrate that ATOH8 is essential for both the differentiation of TSCs into EVTs and the acquisition of their invasive properties, highlighting its fundamental role in placental development and the establishment of a successful pregnancy.

### Context-dependent role of ATOH8 in trophoblast lineage specification

**Fig. 3:**
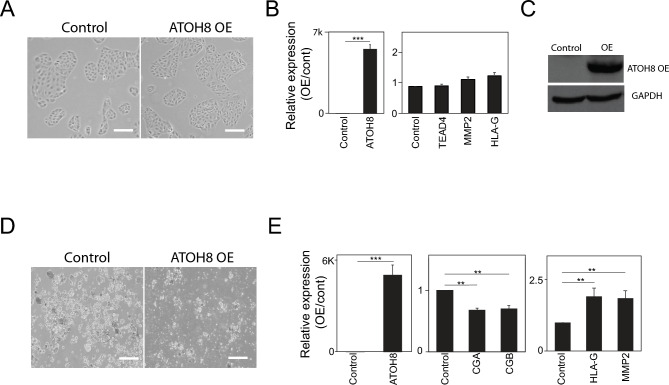
Context-dependent role of ATOH8 in trophoblast lineage specification. (**A**) Bright-field images of control TSCs and TSCs overexpressing ATOH8 (ATOH8-OE). Scale bars indicate 100 µm. (**B**) Relative mRNA levels of ATOH8 and TSC marker genes in ATOH8-OE TSCs compared to control TSCs. Error bars represent mean ± SD from three independent biological replicates. Significance between experimental groups was determined using a two-tailed Student’s t-test. Significance levels of *P* < 0.01 and *P* < 0.005 are denoted by ** and ***, respectively. (**C**) Western blot analysis of ATOH8 in ATOH8-OE TSCs and control cells using a streptavidin antibody. The original blots are presented in the supplementary Fig. 2. (**D**) Bright-field images of control and ATOH8-OE cells following 5 days of differentiation into STs (ATOH8-OE STs). Scale bars indicate 100 µm. (**E**) Relative mRNA expression of ATOH8, ST markers (CGA and CGB), and EVT markers (HLA-G and MMP2) in ATOH8-OE STs relative to control STs. Error bars represent mean ± SD from three independent biological replicates. Significance between experimental groups was determined using a two-tailed Student’s t-test. Significance levels of *P* < 0.01 and *P* < 0.005 are denoted by ** and ***, respectively.

Previous research has shown that the ectopic expression of a single TF can influence cell fate decisions^25^. In this study, we sought to determine whether forced expression of ATOH8 in TSCs is sufficient to induce EVT differentiation, even under conditions that typically promote self-renewal. To test the hypothesis, we utilized the pSBFB vector system^26^ to overexpress ATOH8 in TSCs. Under self-renewing conditions, ATOH8 OE did not result in noticeable morphological changes in the TSCs (Fig. 3A). Although we observed a modest increase in the expression of several EVT marker genes, the overall expression levels were not significantly different from those of control cells (Fig. 3B and C). These results suggest that merely elevating ATOH8 levels in a self-renewing environment is insufficient to robustly trigger EVT differentiation. In contrast, when ATOH8 was overexpressed during the differentiation of TSCs into STs, we observed significantly less aggregation of cells, indicating defects in cell fusion, a critical process for proper ST formation (Fig. 3D). Additionally, this condition was accompanied by an unexpected increase in the expression of EVT marker genes, alongside a decrease in the expression of established ST markers (Fig. 3F). These findings suggest that excessive level of ATOH8 can interfere with normal ST differentiation and may instead promote a partial or aberrant induction of EVT-specific gene programs. In summary, our findings indicate that while ATOH8 OE in TSCs under self-renewing conditions exerts minimal impact on morphology and EVT marker expression, its elevated expression during ST differentiation disrupts normal ST formation and biases cells towards an EVT differentiation program.

### ATOH8 regulates the transcriptional programs and pathways that are crucial for the formation of EVT

**Fig. 4:**
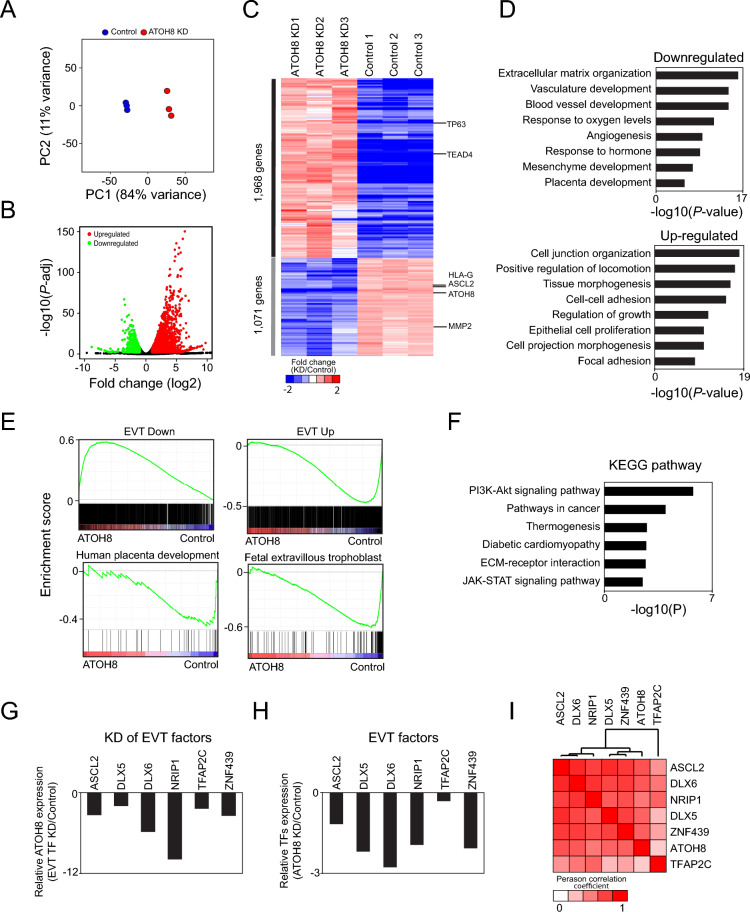
ATOH8 regulates EVT-active transcriptional programs and pathways critical for EVT formation. (**A**) Principal component analysis (PCA) revealing distinct gene expression profiles between ATOH8-KD EVTs and control EVTs. (**B**) Volcano plot highlighting significantly upregulated and downregulated genes in ATOH8-KD EVTs compared to control EVTs. (**C**) Heatmap illustrating differential gene expression between ATOH8-KD EVTs and control EVTs, with key genes such as TSC markers (TP63 and TEAD4), EVT markers (HLA-G, ASCL2, MMP2), and ATOH8 annotated. (**D**) Gene Ontology (GO) enrichment analysis of upregulated and downregulated DEGs in ATOH8-KD EVTs. (**E**) Gene Set Enrichment Analysis (GSEA) indicating impaired activation of gene sets related to EVT activity, human placental development, and fetal EVT in ATOH8-KD EVTs. (**F**) KEGG pathway enrichment analysis of downregulated DEGs in ATOH8-KD EVTs. (**G**) Expression levels of ATOH8 in EVTs with KD of various TFs relative to control EVTs. (**H**) Relative expression of key EVT regulators in ATOH8-KD EVTs compared to control EVTs. (**I**) Pairwise correlation analysis of gene expression profiles following KD of TFs.

To identify the genes regulated by ATOH8 and elucidate their functional roles in EVTs, we performed RNA sequencing (RNA-seq) on both control and ATOH8-depleted EVTs. The RNA-seq reads were aligned to the human transcriptome (GRCh38) from GENCODE (v37) using the Salmon mapper^27^. Principal component analysis (PCA) unveiled distinct clustering between ATOH8-depleted EVTs and control EVTs, confirming significant transcriptomic disruptions due to the loss of ATOH8 (Fig. 4A). Differential expression analysis performed with DESeq2^28^ identified statistically significantly differentially expressed genes (DEGs) between ATOH8-depleted and control EVTs. Using a cutoff of |log2 fold change|> 1 and adjusted *p* value < 0.01, we identified 1968 upregulated and 1071 downregulated genes in ATOH8-depleted EVTs compared to controls (Fig. 4B and Supplementary Table 1). Clustering analysis of these DEGs segregated them into two major gene groups corresponding to up- and down-regulated genes (Fig. 4C). The expression of TSC marker genes, such as TP63 and TEAD4, remained stable, whereas the induction of EVT marker genes was compromised in ATOH8-depleted EVTs.

Subsequent Gene Ontology (GO) analysis was conducted separately for up- and down-regulated DEGs. The downregulated genes in ATOH8-depleted EVTs showed an enrichment for terms related to extracellular matrix organization, blood vessel development, and placenta development (Fig. 4D). In contrast, the upregulated genes were predominantly linked to cell junction organization, cell–cell interaction, and epithelial cell proliferation. These findings indicate that ATOH8 depletion in EVT differentiation diminishes invasive capacity while promoting a more proliferative state. To further delineate the biological processes and pathways affected by ATOH8 depletion, we performed Gene Set Enrichment Analysis (GSEA)^29^. The analysis revealed that ATOH8 depletion not only hindered the induction of EVT-associated genes but also impaired the repression of genes that are typically downregulated during TSC differentiation into EVTs (Fig. 4E). Moreover, the depletion of ATOH8 interferes with crucial processes necessary for human placenta development, particularly in fetal EVTs. The KEGG pathway analysis^30^ of downregulated DEGs highlighted a significant attenuation of the PI3K-AKT signaling pathway in ATOH8-depleted EVTs (Fig. 4F), which is critical for trophoblast survival and invasion^31^.

Notably, ATOH8 operates within a cooperative transcriptional network. Previous studies identified several key regulators of EVT, including ASCL2, DLX5, DLX6, NRIP1, TFAP2C, and ZNF439^11,13^. We found that KD of these EVT regulators resulted in a reduction in ATOH8 expression (Fig. 4G), while depletion of ATOH8 reciprocally decreased the expression of these EVT factors (Fig. 4H). The global transcriptomic profiles of cells lacking an individual TF showed striking similarities (Fig. 4I), indicating a tightly interconnected regulatory circuit. In summary, these findings demonstrate that ATOH8 functions as a central regulator of EVT formation by modulating the expression of EVT-specific genes in concert with other key transcriptional regulators.

## Discussion

In this study, we identify ATOH8 as a critical regulator of human trophoblast differentiation. We mapped the expression patterns and functional roles of ATOH8 across different trophoblast lineages, highlighting its importance in EVT differentiation. We found that ATOH8 is dispensable for the self-renewal of human TSCs, as its depletion did not impact TSC morphology, proliferation, or expression of stemness markers such as TP63 and TEAD4. Supporting this observation, qRT-PCR and scRNA-seq data confirmed minimal ATOH8 expression in undifferentiated TSCs. This indicates ATOH8 is not a core component of the TSC maintenance machinery but becomes functionally critical during lineage commitment. This finding aligns with established paradigms in stem cell biology, where specific TFs are recruited exclusively during differentiation rather than stem cell maintenance^32–34^.

In contrast to its negligible role in TSCs, ATOH8 is essential for the differentiation of TSCs into EVTs. Time-course expression analyses revealed a robust upregulation of ATOH8 specifically during EVT differentiation, while no notable changes occurred during the formation of ST. This highlights ATOH8’s specific role in driving EVT-related transcriptional programs. Depleting ATOH8 severely impacted EVT development, resulting in morphological defects, reduced expression of EVT markers, and decreased invasiveness—key characteristics of EVT function in placental implantation^35^. Notably, ATOH8-deficient cells maintained high levels of TSC marker TEAD4, indicating that ATOH8 is necessary to suppress stemness and activate EVT-specific transcriptional programs during EVT differentiation. The increased expression of ATOH8 in EVTs, confirmed by bulk RNA sequencing, quantitative RT-PCR, and single-cell RNA sequencing, further emphasizes its crucial role in these cells. Interestingly, ATOH8 expression significantly increases after 4 days of EVT differentiation, indicating that ATOH8 may be more critical for EVT maturation than its initiation. Additional studies are needed to elucidate the stage-specific roles of ATOH8 during EVT differentiation.

While ATOH8 is crucial for EVT differentiation, its forced expression in undifferentiated TSCs under conditions that promote self-renewal does not strongly induce EVT markers. This suggests that additional signals are needed to fully activate ATOH8’s regulatory capabilities. It aligns with the notion that transitions in cell fate require well-coordinated signaling and transcriptional regulation^36,37^. Intriguingly, ectopic ATOH8 expression during ST differentiation disrupted cell fusion, suppressed ST markers, and aberrantly induced EVT markers. These context-dependent effects imply that ATOH8 levels must be precisely regulated in both space and time to ensure correct lineage specification, potentially acting as a molecular switch for determining trophoblast fate. Such context dependency is similar to findings in other biological systems, where TFs have lineage-instructive roles only in environments that support these processes^38–40^.

Our transcriptome analysis of ATOH8-depleted EVTs revealed significant changes in gene expression, with 1968 genes upregulated and 1071 genes downregulated. The downregulated genes were enriched in biological processes critical for EVT differentiation, such as extracellular matrix organization, blood vessel development, and placenta development, mirroring the observed invasive and developmental defects. Conversely, the upregulated genes were associated with cell junction organization and epithelial proliferation, reflecting a failure to adopt the invasive, mesenchymal-like phenotype characteristic of EVT.

The diminished invasive capabilities caused by ATOH8 depletion correlate with the downregulation of genes enriched in pathways related to extracellular matrix organization and blood vessel development in ATOH8-deficient EVTs. GSEA confirmed ATOH8’s dual role in activating EVT-specific programs and repressing stemness-associated genes. Additionally, the KEGG pathway analysis highlighted the downregulation of the PI3K-AKT signaling pathway, which is known to regulate trophoblast invasion and placental development^31,41^. This suggests that the PI3K-AKT pathway may act downstream of ATOH8, mediating its pro-invasive effects.

EVTs are crucial for invading the maternal decidua and remodeling spiral arteries to establish the maternal–fetal interface^42^. Our findings emphasize the functional significance of ATOH8 in EVT differentiation. Therefore, it is reasonable to speculate that ATOH8 may be involved in pregnancy-related disorders, such as preeclampsia, where EVT dysfunction is a prominent feature^43^. Future studies should investigate the upstream regulators of ATOH8, its direct transcriptional targets, and its interactions with pathways such as PI3K-AKT. Additionally, the effects of ATOH8 OE should be explored to assess its therapeutic potential in modulating trophoblast differentiation. Comparative studies across species could further clarify whether ATOH8’s role is evolutionarily conserved. In summary, ATOH8 is dispensable for TSC self-renewal but essential for EVT formation. It plays a central role in EVT differentiation and placental development within a transcriptional network that activates invasion-associated programs while suppressing stemness. Its strict lineage specificity and context-dependent activity underscore the precision of trophoblast lineage regulation, ensuring balanced differentiation into STs and EVTs. Further investigation into the mechanisms and clinical implications of ATOH8 is crucial for advancing our understanding of placental biology and pathology.

## Materials and methods

### Cell culture

Human TSCs were obtained from Dr. Takahiro Arima and maintained following established protocols^44^. Cells (CT27) were cultured on collagen IV-coated dishes (5 μg/ml; Corning) in DMEM/F12 basal medium (Gibco) supplemented with 1% ITS-X (Gibco), 0.3% BSA (Sigma-Aldrich), 0.2% FBS (GeminiBio), 0.1 mM β-mercaptoethanol (Sigma-Aldrich), 0.5% Penicillin–Streptomycin (Gibco), 0.5 μM A83-01 (Wako Pure Chemical Corporation), 0.5 μM CHIR99021 (Selleck Chemicals), 0.5 μM SB431542 (Stemcell Technologies), 5 μM Y27632 (Selleck Chemicals), 0.8 mM VPA (Wako Pure Chemical Corporation), 50 ng/ml EGF (PeproTech), and 1.5 μg/ml L-ascorbic acid (Sigma-Aldrich). Cultures were maintained at 37 °C in a 5% CO_2_ humidified incubator with daily medium replacement. At 70–80% confluence, cells were dissociated using TrypLE (Gibco) for 5 min at 37 °C and passaged at a 1:3 split ratio onto fresh collagen IV-coated dishes.

### TSC differentiation into ST and EVT

TSCs were differentiated into STs or EVTs using established methods^44^. For ST differentiation, 2.5 × 10^5^ cells were seeded in 6 cm low-binding dishes containing 3 mL of ST differentiation medium: DMEM/F12 (Gibco) supplemented with 50 ng/ml EGF (PeproTech), 0.3% BSA (Sigma-Aldrich), 0.1 mM β-mercaptoethanol (Sigma-Aldrich), 4% KnockOut Serum Replacement (KSR; Gibco), 2 μM forskolin (Tocris), 0.5% Penicillin–Streptomycin (Gibco), 1% ITS-X (Gibco), and 2.5 μM Y27632 (Selleck Chemicals). The medium was replaced on day 3, and STs were collected on day 5, followed by washing to remove non-viable cells. For EVT differentiation, 7.5 × 10^4^ cells/well were plated in collagen IV-coated (1 μg/ml; Corning) 6-well plates and cultured in 2 mL EVT differentiation medium: DMEM/F12 (Gibco) containing 4% KSR, 0.5% Penicillin–Streptomycin, 0.3% BSA, 0.1 mM β-mercaptoethanol, 1% ITS-X, 7.5 μM A83-01 (Wako), 2.5 μM Y27632, and 100 ng/ml NRG1 (PeproTech). Matrigel (Corning) was added immediately post-seeding to a final concentration of 2%. On day 3, the medium was replaced with NRG1-free EVT medium containing 0.5% Matrigel. By day 6, cultures were transitioned to KSR- and NRG1-free EVT medium with 0.5% Matrigel. Differentiated EVTs were harvested on day 8 for downstream analyses.

### Virus production and ATOH8 knockdown

Short hairpin RNAs (shRNAs) targeting ATOH8 were purchased from Sigma-Aldrich (Supplementary Table 2). 7.3 × 10^6^ 293 T cells were transfected with 2.5 μg of ATOH8 shRNA, 1.7 μg of Δ8.9, and 0.8 µg of VSVG using 15 μl of a GenJet transfection reagent (SignaGen) to generate lentiviruses expressing an ATOH8 shRNA. After 24 h, the medium was replaced with TSC culture medium, and viral supernatant was harvested 48 h post-transfection, filtered with a syringe filter (0.45 µm), and used to infect 2.5 × 10^5^ TSCs/well in 12-well plates. Infected cells were incubated overnight, and the medium was replaced with fresh TSC culture medium supplemented with puromycin to select for the infected cells. Knockdown efficiency and transcriptomic changes were assessed 72 h post-infection.

### Cell proliferation assay

TSC proliferation was quantified using a CCK8 assay (Abcam). Approximately 3 × 10^3^ TSCs, treated with either TCF7L2 shRNA virus or a negative control virus (NC), were suspended in 100 µl of media and seeded in triplicate into 96-well plates. On days 0, 1, 2, and 3, 10 µl of the CCK8 solution was added to each well, followed by a 1.5-h incubation at 37 °C. Optical density was measured at 460 nm using a microplate reader (Thermo Fisher Scientific).

### Generation of inducible ATOH8-overexpressing cells

For doxycycline-inducible ATOH8 expression, TSCs (CT27) were co-transfected with the pSBFB-ATOH8 vector, BirA biotin ligase plasmid, and SB100 transposase plasmid (Addgene, #34,879) using Lipofectamine 3000. Transfected cells were selected with puromycin (1 μg/ml) and G418 (400 μg/ml) for 7 days. ATOH8 expression was induced with 1 μg/ml doxycycline (Fisher Scientific) for 24–48 h prior to experiments. No doxycycline treatment is used as a control.

### Western blot analysis

Cells were lysed in 4X Laemmli sample buffer (Bio-Rad, 1,610,747) containing 2-Mercaptoethanol (MilliporeSigma, M3148) and denatured at 90 °C for 10 min. Proteins were separated by electrophoresis on 10% SDS-PAGE gels and transferred to PVDF membranes (MilliporeSigma, IPVH00010). Membranes were blocked for 60 min at room temperature in 5% skim milk (Bio-Rad, 1,706,404) prepared in TBST. Primary antibodies [GAPDH (Santa Cruz, sc-32233) and Streptavidin-HRP (Cytiva, RPN1231)] were applied overnight at 4 °C. After TBST washes, membranes were incubated with secondary antibody (except for Streptavidin-HRP, which required no secondary due to its HRP conjugate) for 1 h at room temperature, followed by further TBST washes. Proteins were detected using ECL reagent (Fisher Scientific, 45-002-401) and visualized on an iBright CL100 system (Thermo Fisher Scientific).

### Invasion assay

The cellular invasion capacity was assessed using an adapted chamber-based protocol^45^. Briefly, Molecular Probes Secure Seal Hybridization Chambers (Thermo Scientific, S24732) were UV-sterilized for 1 h and mounted onto 12-well plates. A 2:1 (v/v) mixture of Matrigel (Corning) and EVT differentiation medium was polymerized in the chambers for 1 h at 37 °C under 5% CO_2_. Subsequently, 5 × 10^4^ cells suspended in 30 μL EVT medium were seeded into each chamber and allowed to adhere for 1 h in a tilted orientation to promote uniform cell–matrix interaction. Following adhesion, 1 mL of EVT medium was added per well. The culture medium was refreshed on days 3 and 6, following the EVT differentiation protocol. Invasion dynamics were monitored using an EVOS microscope (Thermo Fisher Scientific), with images captured at defined intervals.

### Reverse transcription-quantitative polymerase chain reaction (RT-qPCR)

Total RNA was isolated using the GeneJet RNA Purification Kit (Thermo Scientific, K0731). cDNA synthesis was performed with 500 ng RNA using SuperScript™ III First-Strand Synthesis SuperMix (Thermo Fisher Scientific, 18,080,400), followed by a 20-fold dilution in nuclease-free water. RT-qPCR reactions were conducted in triplicate using PowerTrack™ SYBR Green Master Mix (Thermo Fisher Scientific, A46110) with 2 μL cDNA per reaction. Primers were designed using Primer3 (http://bioinfo.ut.ee/primer3/) and validated for specificity. All primer sequences are provided in Supplementary Table 2. Amplification was performed on a QuantStudio 6 Flex Real-Time PCR System (Thermo Fisher Scientific), with cycle threshold (Ct) values normalized to *GAPDH* as an endogenous control. Relative gene expression was calculated using the 2^(− ΔΔCt) method. In Fig. 1A, we presented gene expression levels relative to GAPDH within each cell type, rather than displaying fold changes between different cell types.

### RNA sequencing and transcriptomic analysis

RNA sequencing was conducted to characterize global transcriptional profiles. Libraries were prepared from 500 ng total RNA using the NEBNext Ultra II RNA Library Prep Kit (NEB, E7770). Polyadenylated mRNA was enriched via oligo(dT) magnetic beads (NEB, E7490), followed by cDNA synthesis with random primers and purification using NEBNext Sample Purification Beads (NEB, E7767). Double-stranded cDNA underwent end repair, adapter ligation (with unique barcodes), and PCR amplification. Libraries were sequenced on an Illumina NextSeq 2000 platform (50 bp paired-end reads). Raw reads were aligned to the human reference genome (GRCh38) using Salmon (v1.10.1)^27^ , with transcript abundance quantified as transcripts per million (TPM) via tximport (v1.18.0)^46^. Count normalization and differential expression analysis were performed using DESeq2 (v1.30.1)^28^, applying the median-of-ratios method. Differentially expressed genes (DEGs) were identified with thresholds of |log2(fold change)|> 1 and adjusted *p* value < 0.01. Other RNA-seq data used in this study were obtained from the published data (GSE212267 and GSE154350).

### Gene ontology analysis

To identify enriched Gene Ontology (GO) terms, Metascape^47^ was used to identify enriched biological processes for DEGs. Enriched KEGG pathways were identified using the DAVID functional annotation tool^48^.

### Correlation analyses

A pair-wise Pearson correlation coefficient between the target genes of two TFs was calculated for each pair of TFs. Clustering analysis and visualization of the data were done with Cluster 3.0^49^ and Java Treeview^50^, respectively.

## Supplementary Information

Supplementary Information 1.

Supplementary Information 2.

Supplementary Information 3.

## Data Availability

All sequencing data we generated were uploaded onto the Gene Expression Omnibus (GEO) with an accession number of GSE293496. The data utilized in this research (GSE293496) is available through the following token: upqzkwuqxditrgx. Additional inquiries regarding the data may be directed to the provided email address: blee6@albany.edu.
