# Supplementary material for: ATOH8 is crucial for the differentiation of human trophoblast stem cells into extravillous trophoblasts
Source: Sci Rep. 2025 Nov 4;15:38582. doi: 10.1038/s41598-025-22484-3 (PMC12586484; doi:10.1038/s41598-025-22484-3)
Supplement: Supplementary file 1 — Supplementary Information 1. [file 41598_2025_22484_MOESM1_ESM.pdf]

Supplementary Figures

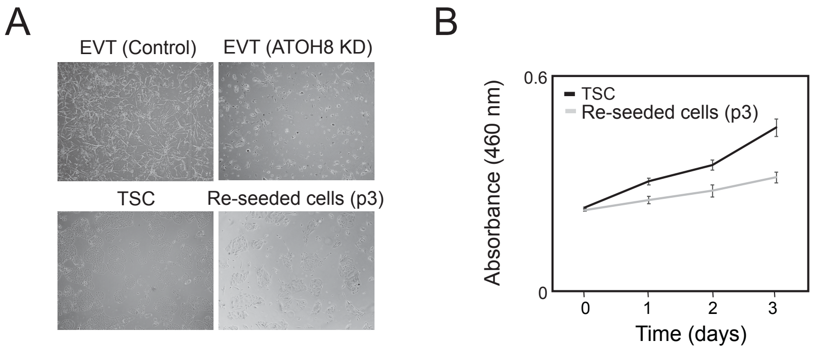

Supplementary Figure 1. Reseeding ATOH8-depleted EVT-derived cells in TSC medium. **A)** Bright-field images of control EVT, ATOH8 knockdown (KD) EVT, undifferentiated TSCs, and cells reseeded from ATOH8 KD EVTs after three passages (re-seeded cells). **B)** Line graph showing proliferation rates of TSCs and re-seeded cells at passage 3 (P3). Error bars represent the mean ± SD from three independent biological replicates.

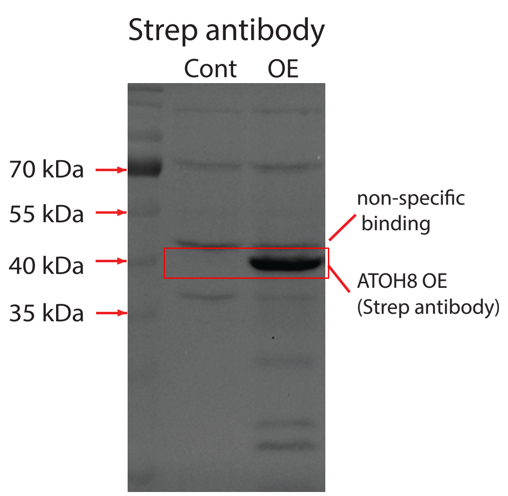

Supplementary Figure 2. Western blot analysis of ATOH8 in ATOH8-OE TSCs and control cells using a streptavidin antibody. The red rectangular outlines the cropped area for Figure 3C.
